# Antibodies and Their Receptors: Different Potential Roles in Mucosal Defense

**DOI:** 10.3389/fimmu.2013.00200

**Published:** 2013-07-16

**Authors:** Rachel E. Horton, Gestur Vidarsson

**Affiliations:** ^1^Institute for Glycomics, Griffith University, Gold Coast, QLD, Australia; ^2^Department of Experimental Immunohematology, Sanquin Research and Landsteiner Laboratory, Academic Medical Center, University of Amsterdam, Amsterdam, Netherlands

**Keywords:** antibody, mucosal, IgA, IgG, IgD, IgM

## Abstract

Over recent years it has become increasingly apparent that mucosal antibodies are not only restricted to the IgM and IgA isotypes, but that also other isotypes and particularly IgG can be found in significant quantities at some mucosal surfaces, such as in the genital tract. Their role is more complex than traditionally believed with, among other things, the discovery of novel function of mucosal immunoglobulin receptors. A thorough knowledge in the source and function and mucosal immunoglobulins is particularly important in development of vaccines providing mucosal immunity, and also in the current climate of microbicide development, to combat major world health issues such as HIV. We present here a comprehensive review of human antibody mediated mucosal immunity.

## Introduction

Mucosal surfaces are the primary point of contact for numerous infectious agents including the world’s three major causes of mortality due to infectious disease, diarrheal diseases, lower respiratory tract infections, and HIV/AIDS. According to WHO statistics, together these account for 27.3% of global deaths. In addition to defending from infection, the mucosal immune system must be able to discriminate between pathogens and foreign proteins derived from ingested material in order to prevent potentially harmful responses to innocuous antigens.

Initial defense occurs through indiscriminate mechanical action, mucus, cilia, and the epithelial cell barrier, for example. The epithelial cells themselves provide these surfaces with bactericidal proteins and antimicrobial peptides (1) and take a large part in generating the cytokine milieu required for the adaptive immune response, and probably participate directly in the initiation and eradication of infection as discussed below. More specialized action is directed and regulated by surveillant myeloid phagocytes and the other cells of the mucosal immune system, located in local lymphoid compartments which make up the mucosa-associated lymphoid tissue (MALT) as well as in the lamina propria. Cellular immunity has a clear role in induction and coordination of the adaptive immune response at mucosal surfaces [reviewed in (2)] but here we concentrate on the contribution of the end product of the humoral immune response. This is characterized by secretory IgA (SIgA) that is present at higher levels at mucosal sites than other immunoglobulins, notable exceptions to this rule being the female and male genital tract (3–5), bronchoalveolar fluid, and bile (6) where IgG is dominant. Although present at lower levels in external secretions [levels of all immunoglobulins in mucosal secretions are reviewed by Norderhaug et al. and Mestecky et al. (5, 7)], IgM also has a role in mucosal defense and it has recently been noted that IgD may play an important part (8).

It has been observed that macromolecules derived from plasma can exude to the mucosal surface by bulk flow through epithelial tight junctions that can filter these molecules depending on size (∼7–15 Å) and sub-epithelial hydrostatic pressure (9, 10). However, the different immunoglobulin isotypes (IgG ∼55Å), are bigger than most of these tight junctions to allow for free passage. They do however interact with novel immunoglobulin receptor systems that mediate their functions via passive transfer, active destruction through phagocytosis, or antigen sampling and presentation for enhanced immune responses. Collectively, these functions are crucial for the interplay between the innate and adaptive immune systems. Current knowledge in this area together with latest findings on how subversive pathogens evade these mechanisms are reviewed below.

## Sources and Passive Function of Mucosal Immunoglobulins

### Immunoglobulin A

**Figure 1:**
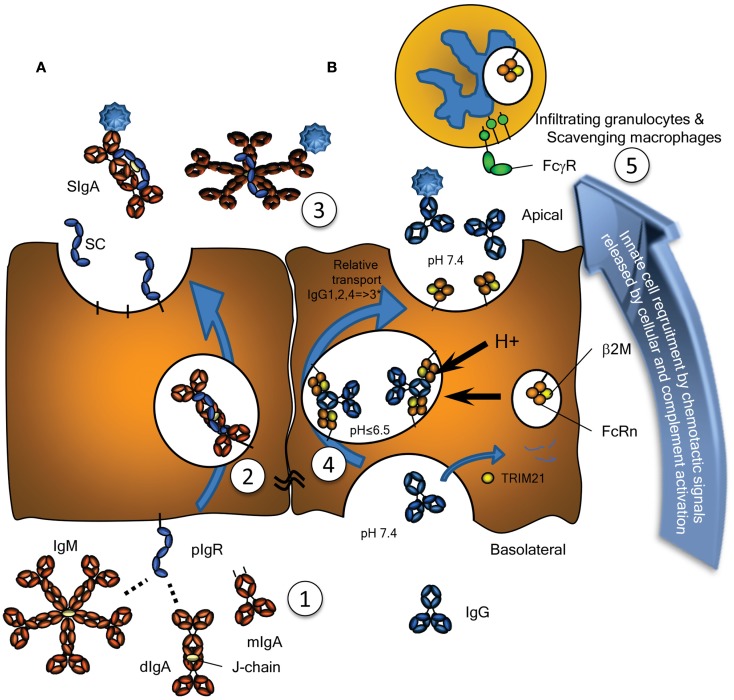
**Transport of Immunoglobulins to mucosal surfaces**. Mucosal IgA, IgM, produced locally at the lamina propria, and IgG, produced either locally or systemically, are transported by **(A)** the Polymeric Immunoglobulin Receptor (pIgR) or **(B)** by the Neonatal Fc-Receptor (FcRn), respectively. **(A)** The pIgR, expressed on serous-type secretory epithelial cells, specifically binds J chain containing dimers (and larger polymers) of IgA or pentamers of IgM at basolateral surfaces, but not serum-derived monomeric IgA (1). This prompts uptake and transport through the cell (2), eventually resulting in subsequent vesicle fusion at apical sites where the pIgR is cleaved, releasing the extracellular domain of pIgR either as free SC (unoccupied) or bound SC in SIgA and SIgM (3). SC remains bound to the IgA and IgM, for IgA covalently, blocking its interaction with the FcαRI, but can also be released from IgM upon purification. **(B)** Mucosal transport of IgG is initiated by pinocytosis and/or receptor mediated uptake of IgG. During the initial stages of IgG-transport, the pH is probably neutral, under conditions where FcRn has no or negligible affinity for IgG (1). After acidification of the developing vacuoles and fusion with FcRn-containing vesicles or tubules, changes in the charge of the IgG-Fc tail induce the recognition of IgG by FcRn, where a single IgG is probably recognized by two FcRn molecules on parallel membranes (4). This induces the rescue of this IgG from lysosomal degradation and transcytosis to the apical surface, where the cargo is released at physiological pH (3). The relative transport rate for the IgG subclasses is similar to what is seen for their FcRn-mediated half-life, with few exceptions, as discussed in the text, and likely to be allotype dependent for IgG3 (indicated with an asterix). Interaction of opsonized pathogens or immune complexes may also cause local complement activation, eventually leading to target lysis and/or opsonization, but also release of C5a, a powerful anaphylatoxin and a chemo attractant. Interaction with myeloid FcγR and FcαR also leads to degranulation and release of chemotactic factors, thereby inducing enhanced migration of lymphoid and myeloid cells to the inflamed site, here depicted above the surface, but in real life probably scavenging the surface by close adherence (5), eventual clearance of the infection and resolution of inflammation. As discussed in the text, these immunoglobulin transport pathways may also neutralize pathogens localized either at the basolateral side or within intracellular vacuoles allowing for their intracellular degradation or expulsion. Also discussed in the text, is the possible involvement of the cytoplasmic Ig-receptor TRIM21 for intracellular degradation of opsonized non-enveloped viruses and intracellular bacteria.

The mucosal environment is programed to induce B cell class switching to IgA production as both mucosal T cells and mucosal epithelial cells themselves produce TGF-β and IL-10, cytokines essential for programing of committed IgA producing B cells (11). Although, systemic IgA responses tend to occur in germinal center reactions, and require T cells, a significant portion of the IgA response (CD27^−^IgA^+^) does not require T cells, as they harbor low frequency of somatic hypermutations and develop normally in both mouse and humans lacking either CD40 or CD40L, respectively (12, 13). In the gut, this T cell independent mechanism preferentially leads to class switching to IgA2 with λ-light chains (13, 14). The reason for the selective usage of the λ-locus for the light chains is unknown, but may reflect the selection for binding to unknown human pathogens – a feature also found for IgD responses in tonsils (8). Although serum IgA is mainly monomeric, originating from the bone marrow, but also lymph nodes and spleen, at mucosal surfaces it is usually polymeric, synthesized locally by plasma cells located in the lamina propria (15). These polymers, most often dimers, linked by the cysteine rich J chain, are secreted across the mucosal epithelium via the polymeric immunoglobulin receptor (pIgR, also termed membrane secretory component, SC). Post transcytosis protease cleavage releases IgA complexed with the extracellular part of the pIgR (bound SC), as SIgA into the mucosal lumen (Figure 1A). SIgA has traditionally been perceived as an anti-inflammatory mediator (16) with three major functions: (1) to physically block pathogen attachment and invasion (immune exclusion), (2) to recognize foreign antigens and escort them through epithelial cells ridding the mucosa of excess antigens, and (3) to intercept viruses intracellularly (during transcytosis), thereby facilitating their neutralization and expulsion already within infected cells (17). This latter mechanism was suggested and experimentally confirmed in the early 90s and has been reconfirmed for the measles virus (17, 18). Further support for this view of SIgA as a non-inflammatory antibody comes from its poor ability to activate the classical complement pathway; it lacks the C1q binding motif found in IgG. Although it has been shown to activate complement through the lectin pathway, this is probably dependent on the glycosylation status of in the IgA1 (19). IgA1 glycosylation has recently been shown to be highly complex, with several glycosylation isomers (20, 21), suggesting that IgA-function may also be modulated though glycosylation in a similar manner as recently described for IgG as discussed below (22). Surprisingly, glycosylation of the IgA-bound SC has also been reported to affect binding of IgA to commensal bacteria (23). Importantly, IgA can also actively mediate protection against invasive disease, because, in addition to transport of secretory immunoglobulins, the pIgR is able to transport immune complexes across polarized cell lines *in vitro*, releasing them at the apical surface, hence suggesting a direct role for the pIgR and IgA in clearance of pathogens from the sub-epithelial mucosa (24).

### Immunoglobulin M

IgM is the first immunoglobulin to appear during an infection and is found at detectable albeit low levels in secretions. The largest of the antibodies, it occurs as a pentamer and is transported across epithelial cells in the same way as IgA, via its J chain and the pIgR (Figure 1A) (25). There are fewer IgM than IgA secreting cells at mucosal surfaces, and its net-mucosal transfer is mediated less efficiently than that of IgA (25). As pIgR-mediated epithelial transport of both isotypes occurs at equal rates *in vitro* (5), the reason for the lower transport is probably due to its large size and therefore less efficient diffusion through epithelial basement membranes, resulting in a lower mucosal concentration than what could be predicted from the local production of IgM. Despite these low mucosal levels, it does show elevated levels in individuals with IgA deficiency, in a compensatory manner, and provides some protection from infection. Although it hardly activates myeloid cells, mediating effector functions primarily through complement, a potential role has recently been described for the myeloid IgM receptor, TOSO (26). Mice lacking this receptor show elevated Reactive Oxygen Species (ROS) production after formyl-Methionyl-Leucyl-Phenylalanine (fMLP) stimulation, but reduced IgM-mediated phagocytosis, reduced inflammatory cytokine production after challenge with *Listeria monocytogenes*, ultimately leading TOSO^−/−^ mice to succumb faster to *Listeria* infection (27). The role of this receptor in humans for IgM-mediated antimicrobial defense remains to be elucidated.

### Immunoglobulin D

Monomeric IgD forms the major part of the B cell receptor and is therefore present in membrane bound form on naïve and memory IgM^+^IgD^+^ B cells and also on class switched IgM^−^IgD^+^ memory B lymphocytes (8). IgD secreting plasmablasts are scarce in bone marrow and the digestive system (28), but are found at higher frequencies in the lacrimal gland, nasal mucosa, and tonsils (29), with as many as 20–25% of plasmablasts/plasma cells producing IgD being reported for the tonsils (8). The number of these cells has however been disputed, and may be on average be below 5% (30). Research concerning the function of IgD has lagged behind that of other immunoglobulins, due in part to methodological difficulties in detection, its low concentration in serum, and its absence in a number of animal systems including rabbit and guinea pig (31). However, human IgD class switched B cells, most of which also express the λ-light chains as discussed above for IgA, have recently been identified and shown to secrete both mono- and poly-reactive antibodies which react with respiratory pathogens including *Haemophilus influenzae*. It has been hypothesized to be part of an evolutionarily conserved immune surveillance system activating effector functions of basophils (8). Many questions regarding the function of IgD, including how IgD finds its way into secretions – which may occur by either receptor- or non-receptor mediated mechanisms, remain unanswered [reviewed in (32)], and await confirmation.

### Immunoglobulin G

The presence of IgG in external secretions has largely been ignored in the literature, but recent work has demonstrated that this isotype is capable of mediating active humoral protection in various mucosal locations (33–36). Human IgG consist of four subclasses, IgG1, IgG2, IgG3, and IgG4, with reference to their decreasing abundance in serum. They all have remarkably different effector functions, with respect to both complement activation and binding to FcγR (as discussed in a more detail below), with the general order of activating capacity for both being IgG3 > IgG1 >> IgG2 > IgG4. IgG is present at significant levels at all mucosal surfaces, and although IgA is the most abundant mucosal antibody, IgG concentrations actually exceed those of IgA at a number of locations, including the male and female genital tracts and bronchoalveolar fluids, where it is the predominant antibody isotype (3, 4). Mucosal IgG is believed to be produced locally as IgG producing plasma cells are certainly found locally in the genital tracts of both sexes, but can also transudate from serum – the level of which is under regulation by hormonal- and menstrual-cycle in females (4, 37). Regardless of the source, IgG is probably transported through all epithelial layers, under conditions when the epithelial layer is intact with low sub-epithelial pressure, by the neonatal Fc-receptor (FcRn), a MHC class I homolog (Figure 1B). Parenteral administration of passive neutralizing IgG has been shown to prevent oral rotavirus (36), oral and vaginal HIV-1 transmission (34), and lung infection by *Streptococcus pneumoniae* (35). The mechanisms of this protection may be different depending on the site of action, but have been proposed to occur in secondary lymphoid tissues, mediated by active phagocytosis. Pathogen clearance may also involve complement (35) and it is possible that strong activation of complement by IgG could cause inflammation and damage to the epithelial barrier. Cross-linking of Fcγ receptors also triggers a range of other effector functions including phagocytosis, respiratory burst, and Antibody Dependent Cell-mediated Cytotoxicity (ADCC) processes that release inflammatory mediators and may also cause damage to epithelial barriers in chronic inflammation [reviewed in (38)]. The activity of the IgG response, can be modified through addition and removal of glycan-moieties at Asn297 in the Fc-portion [reviewed in (39)]. In particular core-fucosylation, normally present is serum of>90% of all IgG, affects the binding of all IgG subclasses to FcγRIIIa/b up to several orders of magnitude with accompanying increases in cellular responses (40). Importantly, this type of glycosylation can be regulated at the level of the B cells in humans, as it can be found in certain responses, e.g., anti-platelet responses seen in pregnancy (22). A role for this type of regulation during mucosal immune responses still needs to be investigated.

IgG subclass levels found at mucosal sites, with relative low IgG3 concentrations compared to plasma, correspond what is known about half-life extension (long half-life of IgG1, IgG2, and IgG4, but short half-life of IgG3) and transport though the placenta (no active transport of IgA, but active transport of all IgG, of which transport of IgG1, and IgG4 exceed that of the mother, but with low transport of IgG3 and IgG2), both roles carried out by the FcRn (41, 42). Mucosal transport of IgG subclasses therefore correlates with their known half-life and placental-transport properties, suggesting IgG to be actively transported across these mucosal surfaces by FcRn. For example, mucosal transport of IgG3, the only IgG subclass with a half-life of only 1 week (compared to 3 weeks for the other subclasses) seems invariably lower than for the other subclasses (43). A potential concern is that it has been proposed that the long hinge of IgG3 may be more susceptible for proteolytic cleavage (33), but given that this effect is found, for example, in seminal secretion and in saliva, which are quickly expelled, this seems less likely (43). Transport of IgG2, with the exception of salivary transport, seems to be reduced, mirroring what is seen for placental-transport – where both IgG2 and IgG3 are transported to a lesser degree than IgG1 and IgG4 (44). Although mucosal transport of IgG3 was much reduced compared to the other subclasses in the above mentioned study and others (43), we recently found IgG3 is never the less very efficacious in protecting against lung infection by *S. pneumoniae* (33). More importantly, the relative abundance of mucosal IgG3 may also be increased in individuals expressing the G3m(s,t) allotype of IgG3 (common in Asians). This is because, unlike the G3m(b) or (g) allotypes (common in Europeans) with an arginine at position 435, the G3m(s,t) allotype contains an histidine at this position. This results not only in improved pH-dependent binding to FcRn and prolonged half-life, but also increased placental-transport, suggesting mucosal transport of this IgG3 allotype to be increased as well (33, 41). As such, this allotype of IgG3 with both prolonged half-life and even stronger effector functions than all the other IgG subclasses (both in terms of complement activation and binding to FcγR), may prove important in both health and inflammatory diseases at the mucosa.

## Receptors and Adaptive Immunity

There are two main, and subsequently well characterized IgA receptors, the pIgR and FcαRI. Several other receptors have also been reported to bind IgA with, as yet, unclear or uncharacterized functions. The receptors for IgG can be classified into the well-known FcγR family, consisting of several proteins expressed on myeloid cells, and the FcRn that is ubiquitously expressed at various levels in different cells. These and other less characterized immunoglobulin receptors are summarized in Table 1 and discussed below.

**Table 1:** **Immunoglobulin receptors involved in transport and/or functions of effector cells or molecules at epithelial surfaces**.

Receptor	Ligand	Cell type	Characteristics
pIgR (102)	J chain in the context of IgA or IgM	Secretory epithelial cells	Transports IgA/IgM across epithelial cell layer
FcαRI (53, 103)	IgA1/2 (SIgA)	Myeloid cells	Major IgA receptor*
Fcα/μR (104)	IgM > IgA	B cells, macrophages	Type I transmembrane protein, mediates B cell endocytosis of antibody coated targets
FcμR/TOSO (26)	IgM	B- and T-cells	Highly specific for IgM. Currently undetermined function
β-galactosyltransferase (105)	pIgA, mIgA, SIgA	Liver, myeloid, intestinal epithelial cells	Unknown function, cation independent binding
Transferrin receptor (CD71) (106)	mIgA1 > pIgA	Renal mesangial cells	Unknown function, binding of IgA does not interfere with transferrin binding
SC-receptor (107, 108)	Secretory component	Eosinophils, basophils	Binding of IgA may cause degranulation
Asialoglycoprotein receptor (ASPGR) (109)	Terminal galactose and *N*-acetyl-d-galactosamine residues	Liver, myeloid, epithelial cells	Involved with IgA clearance from blood and liver
DC-SIGN (92, 94)	IgA/IgG	Sub-mucosal dendritic cells	Possible involvement in immune surveillance at mucosal surfaces, immune regulation
FcγRI	IgG	Monocytes, neutrophils, macrophages	These are the three major IgG receptor classes. A number of further isoforms have been described, of which all, except FcγRIIb, mediate cellular activation
FcγRII	IgG	Monocytes, neutrophils, eosinophils, basophils, B cells, platelets, macrophages, langerhans cells, endothelial cells of the placenta	Different functions are meditated on cross-linking by ligand or specific antibody, including phagocytosis, ADCC, cytokine release, superoxide production, and antigen presentation, except for the FcγRIIb which inhibits these ITAM-responses though its ITIM-signaling encoded in its cytoplasmic tail (110, 111)
FcγRIII	IgG	Monocytes, neutrophils, eosinophils, NK cells, T cells, macrophages, kidney mesangial cells, placental trophoblasts	
FcRL5 (and FcRL4) (112)	IgG (FcRL4 only IgG3 and IgG4)	B cells (FcRL4 on memory B cells)	ITIM-containing inhibitory receptors, that probably functions similar to FcγRIIb
TREM21 (98, 99)	IgG, IgM, others?	Mostly cells, high expression in myeloid cells	Cytoplasmic receptor, prompting ubiquitination-depended breakdown of IgG-opsonized particles
FcRn (64, 70, 72, 113)	IgG	Ubiquitous, high in epithelial cells, placental syncytiotrophoblasts, endothelial cells, monocytes, PMNs, dendritic cells	Transplacental transport, transepithelial transport, IgG regulation
FcδR (8)	IgD	Basophils, mast cells	Cytokine inducible. Other, less defined receptors have also been described on lymphocytes and basophils (32, 114).
TRIM21 (99, 101)	IgG, IgM, IgA	All cells, high on immune and endothelial cells	Intracellular Ig-receptor targeting cytoplasmic Ig-complexes for ubiquitin-dependent proteasome degradation
FcεRII (CD23)	IgE	B cells, enterocytes (90, 91)	In the gut CD23 promotes bidirectional transport of IgE and IgE complexes in the gut, thereby providing antigen sampling mechanism, suggesting a role for food allergies, and possibly protection against helminth infection

*A number of even less established receptors for IgA on mesangial-, M-, epithelial-, and T-cells have also been reported.

### Polymeric immunoglobulin receptor

The pIgR is responsible for the transport of both SIgA and IgM from the basolateral to the apical epithelial surface at the mucosa (Figure 1A). Irrespective of being bound by IgA or IgM, upon the arrival of the pIgR at the apical surface, the extracellular SC domain is cleaved and the remaining receptor degraded. The SC remains attached to IgA and IgM, but for IgA the linkage is more stable as it is covalent (45). Therefore free, soluble SC can be found at the mucosal surface and each pIgR molecule can only mediate one cycle of transport (46). As discussed below, the pIgR may also recycle without cargo and without cleavage of the SC. Upregulation of pIgR synthesis is mediated by several cytokines including IFNγ, IL-1, and TGF-β, resulting in increased transport of IgA across the epithelial layer (47, 48). Recently, secretion of IL-17 from Th17 cells was suggested to not only stimulate homing of B cells to the lungs but also to upregulate pIgR expression and enhanced secretion of IgA and IgM, a pathway which is usually held in check by antigen-specific by regulatory T cells (49). Thus, under inflammatory situations or general immunological imbalance, the resulting Th17 like conditions may stimulate mucosal IgA and IgM production and secretion, and intensify the response.

### FcαRI

**Figure 2:**
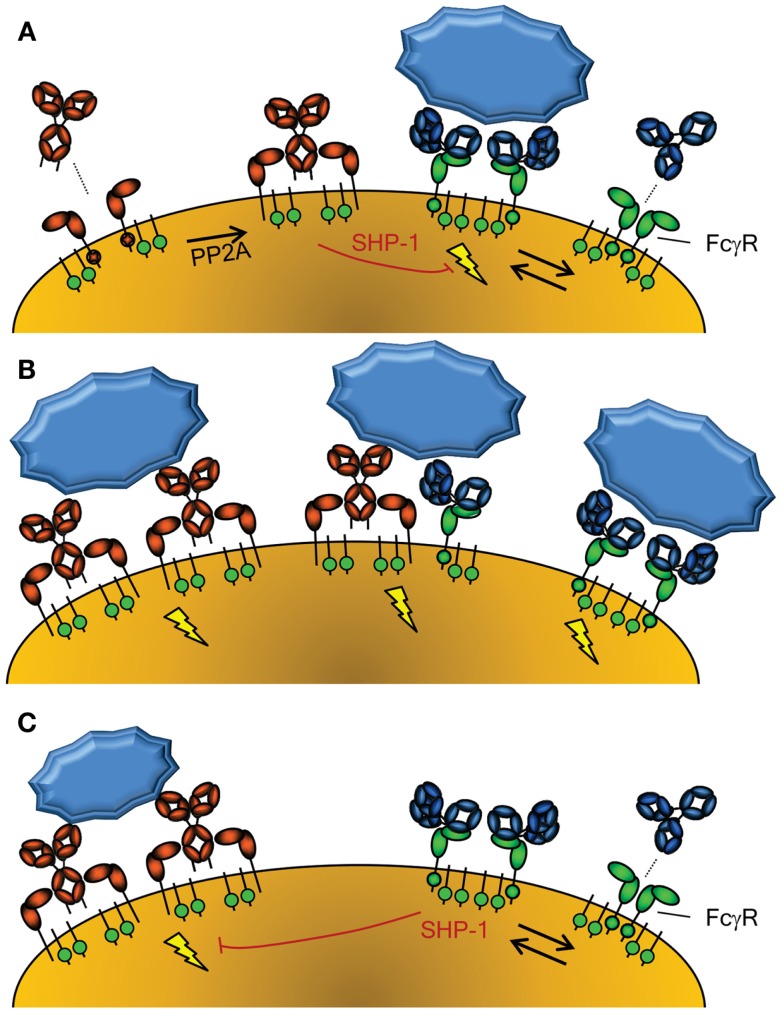
**Activation of cellular responses through Fc-Receptors**. **(A)** The activity of FcαRI (left) is controlled through inside-out signaling through PP2A, that dephosphorylates the intracellular tail of FcαRI, enabling binding of IgA (58). Conversely, FcγR (right) seem to be continuously enabled, although crystallographic data suggest a dimeric form may exist that cannot interact with IgG without disrupting the inert FcγRIIa dimer (115). The FcγRIIa engaged by IgG may however form a higher order dimer, or multimer, with either another FcγR-IgG unit or unligated FcγR, forming an active signaling complex after engagement with IgG-opsonized target. However, without crosslinking of FcαRI through IgA and it’s cognate antigen, the FcαRI has been reported to lead to down regulation of FcγRI signaling through phosphorylated SYK (58). **(B)** Importantly, co-engagement of FcγR and FcαR results in a strong activation of phagocyte responses, with FcαRI leading to a more prominent respiratory burst activation, while FcγR result more a prominent phagocytosis response (54). **(C)** FcγR can also mediate inhibitory signal, because IgG-ligated, either by monomers at high concentrations, dimers, or F(ab’)_2_-anti FcγR, can also cause inhibition of other ITAM-, but also non-ITAM, depended cellular activation, also through phosphorylated SYK (60).

The other major IgA receptor is the FcαRI which is found constitutively on cells of myeloid lineage although expression levels are affected by cytokines and vary with cell type (50). The FcαRI is found in association with the common FcR γ-signaling chain (also found associated with FcγR) which has a cytoplasmic immunoreceptor tyrosine-based activation motif (ITAM) (51). The FcR γ-chain is responsible for initiation of intracellular events and effector functions, including inflammatory responses against IgA opsonized pathogens. FcαRI activation status can be modulated through protein phosphatase 2 (PP2A) dependent inside-out signaling, whereby proximal inflammatory mediators may determine whether or how FcαRI is spatially integrated into the lipid membrane and therefore if FcαRI is capable of interacting strongly with IgA to activate cellular responses (Figure 2A) (52). In contrast to serum-derived monomeric IgA (mIgA), which can mediate a number of inflammatory effector functions through the FαRI including endocytosis, phagocytosis, a particularly strong respiratory burst, and ADCC (50, 53, 54), SIgA is unable to interact with FcαRI. This interaction with the FcαRI is blocked by steric hindrance of the bound SC, contributing to the anti-inflammatory role of IgA at mucosal surfaces where SIgA accounts for the majority of IgA present (50, 53–55).

### FcγR

The major IgG receptors, FcγRs, are represented by three different classes found on cells of myeloid origin. They activate myeloid cells through the same ITAM-dependent FcRγ-signaling chain (except for FcγRIIa and FcγRIIc that have their own cytoplasmic ITAM-motives, and FcγRIIb with an immunoreceptor tyrosine-based inhibitory motif, ITIM) upon receptor crosslinking. This results in similar effector functions as seen for the FcαRI that can be down modulated through a negative feedback loop via the inhibiting FcγRIIb [extensively reviewed elsewhere (56)] (Table 1). The binding affinity to each of the FcγR differs markedly between different IgG subclasses, the general consensus being IgG3 ≥ IgG1 > IgG2 > IgG4, with few exceptions depending on the FcγR. The most important exceptions from the rule are that IgG2 only binds FcγRIIa, and preferentially the H131-FcγRIIa allotype (also known as the low-responder form, based on its low binding to mouse IgG1) and IgG4 binding FcγRI with considerable high affinity. Remarkably, the affinity of the IgG subclasses are not directly related with binding of myeloid cells to opsonized targets, as IgG3 seems to surpass all other subclasses in this respect for all FcγRs, with few exceptions (57).

### Crosstalk between activating FcR families

In the absence of specific antigen recognized by mIgA, mIgA is also able to mediate anti-inflammatory functions by interacting with FcαRI at a low level in a monovalent fashion (without crosslinking FcαRI) and impeding ITAM-initiated signaling of FcγR, by recruitment and phosphorylation of SHP-1, probably through the low level activation of SYK (58, 59) (Figure 2A). In contrast, serum IgA invokes a massive activation of the myeloid system when opsonized antigen-specific IgA is present (50, 53, 54) (Figure 2B). Thus IgA has several roles; while SIgA is generally anti-inflammatory, mIgA, through FcαRI, can either mediate massive activation of the myeloid system (Figure 2B) or be immunomodulatory (Figure 2A), depending on the presence or absence of its cognate antigen.

Similar to the FcαR, FcγR, human FcγRIIIa can mediate inhibitory responses of other cellular activation responses, including fMLP, uptake endocytosis through the MARCO scavenging receptor, but also other ITAM bearing receptor, like FcεRI (60) (Figure 2C). A similar mechanism has been demonstrated in mice for mouse FcγRIII (61). This inhibition also involves recruitment of SHP-1, and its phosphorylation, probably through SYK. This inhibition, both for FcγR and FcαRI, therefore seems to be a general phenomenon of ITAM mediated signaling, that acquires inhibitory ITAM signaling properties (ITAMi) with low level of engagement through monomer or dimers, but activation properties only when fully cross linked (58–60). This pathway may thereby explain at least partly the inhibitory effect of high-dose Intravenous Immunoglobulin (IVIg) treatment in autoimmune diseases. But what would be the physiological relevance of this inhibitory mechanism, as most immune responses against invading pathogens result not only in either IgA or IgG responses, but both? Perhaps the solution is to be sought by the fact that some autoimmune responses seem to be mostly restricted to IgG, allowing for dampening of these unwanted responses. If true, it may also explain increased tendency of autoimmune diseases, for example in IgA deficiency (62).

### Neonatal Fc-receptor

The neonatal Fc-receptor is responsible for the materno-fetal transplacental-transport of IgG (63), and the extension of the half-life of IgG and albumin (64). Originally, FcRn was described in rodents to transport IgG from mothers milk to the suckling neonates, providing them with humoral immunity after birth (65, 66). Later work utilizing electron tomography, has shown IgG to be taken up by FcRn-positive epithelial cells in the proximal small intestines, where it traverses the cell through entangled network of tubular structures, through multivesicular bodies, accumulating in basolateral intercellular spaces separated from the gut lumen by tight junctions (67, 68). Initiation of transport, takes place after binding of IgG and albumin by FcRn at low pH, which results in the rescue of molecules from developing lysosomes. The receptor then transports its cargo to either the apical or basolateral surface, and releases it at neutral pH, thereby accomplishing transcytosis or recycling. In this way, FcRn is apparently able to transport IgG effectively, even through stratified cell layers as for example in the placenta [reviewed in (69)], and perhaps also through walls of vaginal and oral cavities, although this has not been proven. However, the FcRn is not only expressed on epithelial and endothelial cells, but also at high levels on myeloid cells where it participates in phagocytosis of IgG-opsonized bacteria and immune complex-uptake (70) and can enhance antigen presentation on dendritic cells (DCs) (71– 77). It has also been reported to be responsible for bidirectional transport of IgG across epithelial layers and to play a vital role in delivery of IgG to the lumen of the gut, genital areas, and lung (73, 74, 78) (Figure 1B). In this way, FcRn has for example been shown to bring protective immunity against helicobacter species (79). Inflammation caused by such infection, may affect FcRn expression, as FcRn is sensitive to NFκB-signaling. In accordance, FcRn expression is upregulated by TNF-α, but also by the TLR ligands gram negative endotoxins (lipopolysaccharides) and CpG oligodeoxynucleotides (80). This may not only cause increase in the FcRn-saturation levels, causing increase in systemic IgG and albumin levels (64, 81), but also increased transport of locally and/or systematically produced IgG and albumin, and perhaps explain increased mucosal transport of IgG in pIgR KO mice that display increased IgG and albumin levels (82). The increased salivary IgG levels found for example in excessive Gingivitis cases, may therefore very well due to FcRn-upregulation, but it still need to be tested whether this is the case, as inflammation may also cause excessive leakiness of the epithelium (83).

**Figure 3:**
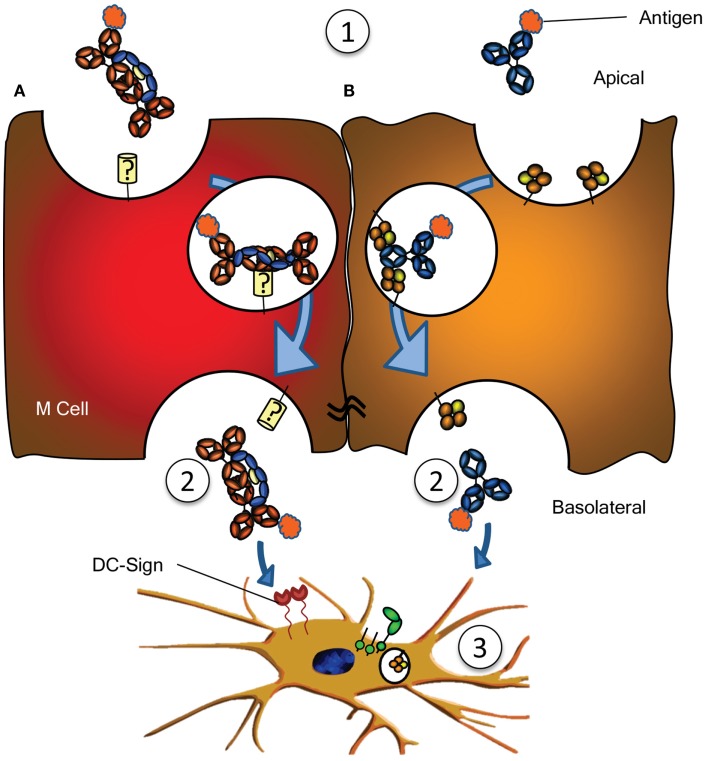
**Antigen sampling and amplification of humoral immune responses**. **(A)** SIgA recognizing its cognate antigen (1) is taken up by M cell, possibly though a yet unidentified receptor on M cells. This complex has been demonstrated to traverse the epithelial layer (2) and to be taken up by DCs (3) possibly through DC-SIGN recognition. **(B)** Similarly, antigen bound by IgG (1) can traverse the epithelial layer through FcRn-depended transcytosis and taken up by DCs through FcγR and FcRn (2) (73). Both IgG- or IgA-mediated antigen uptake can enhance antigen presentation, subsequent activation and proliferation of antigen-specific B- and T cells, boosting immune responses (3). See text for more details.

In addition to IgG catabolism and transfer, the FcRn may mediate transport of IgG-antigen complexes from the basolateral surface back to the intestinal lumen. In this way FcRn has also been demonstrated to actively participate in the intracellular viral neutralization (84). Perhaps even more importantly, this transport is bidirectional as FcRn can mediate antigen sampling by transporting these IgG-antigen complexes from the lumen to regional lymphoid structures for amplification of immune responses (74) (Figure 3). The IgG response generated in this way further stimulates secretion of immunoglobulin and its transport to the lumen where it actively mediates clearance and/or protection against pathogens, as recently shown for immunity against HIV and *Citrobacter rodentium* in a mouse models (74, 85, 86). FcRn is therefore active at various levels in immunity; from half-life extension of IgG to transport of IgG to relevant sites, and together with classical FcγR, directs pathogen eradication by phagocytosis and amplification of immune responses (70, 73–77, 85).

In rodent models, FcRn also has a potentially important role in transmission of IgG from the breast milk of allergic mothers which has been shown to decrease the severity of allergic disease in the suckling offspring (87–89). This pathway is probably limited to species where materno-fetal transfer of IgG takes place through breastfeeding, and is thus not applicable to humans where IgG is almost exclusively transferred to the neonate before birth via the placenta. However, it is as of yet unknown if placental-transport of IgG may affect sensitization of the newborn to allergens.

### Less characterized Ig-receptors

Numerous other receptors have been described to bind immunoglobulins (Table 1), but many need confirmation, while the function of many others is completely unknown. An example is the FcεRII (CD23), which has been described to translocate IgE bilaterally through gut epithelial cells, capable of translocating IgE-bound allergens, perhaps explaining some aspects of food allergy, but also immunity against gut parasites (90, 91). One intriguing scenario is the possible role for SIgA in immune surveillance by the sampling of antigens from the lumen of the intestine. By transcytosis through M cells of the Peyer’s patches on its own or in complex with antigen, IgA has been found to interact with sub-epithelial DCs. The receptor on M cells is hitherto unreported but interactions with DCs are believed to be mediated by DC-SIGN (Figure 3A) (92). Curiously, DC-SIGN has also recently been described to bind Fc-sialylated IgG (93, 94), a subfraction of IgG, representing approximately 10% of all IgG. Although the interaction between sialylated IgG and DC-SIGN in this case was found to have immunomodulatory properties (given as IVIg in rodent models), up regulating the inhibitory FcγRIIb in the myeloid compartment, the normal physiological role of this interaction between Ig and DC-SIGN is still completely unknown. Curiously, DC-SIGN is encoded in a cluster of related genes, which includes CD23, which, like DC-SIGN, is a type II trimeric membrane lectin. The C terminal domain of these proteins, in CD23 encoding for the binding domain for IgE – to which it binds through protein–protein interaction (95) – share 42% sequence homology, and are structurally highly conserved (structural alignment of DC-SIGN PDB ID 1K9J vs. CD23 PDB ID 4EZM). Furthermore, the location in where CD23 interacts with IgE, is highly homologous to CH2/CH3 of the IgG-Fc, suggesting that DC-SIGN may perhaps also interact with IgG through protein–protein interactions (96), which would explain why a recent study found no indication of DC-SIGN interacting with glycan structures of IgG (97). An even more curious receptor, the Tripartite Motif-containing 21 (TRIM21) is expressed in the cytoplasm of most cells, particularly in immune cell, and recognizes at least both IgG and IgM (98, 99). The binding to IgG is of very high (*K*_d_ = 37 nM), requires the TRIM21 PRYSPRY motif, and binds IgG in the interface of CH2 and CH3, to a similar same interface as protein A/G and FcRn (99, 100). The peculiar cytoplasmic location of TRIM21 renders it physically incapable of interacting with immunoglobulin under normal circumstances. However, TRIM is apparently capable of recognizing opsonized viral targets after internalization and cytoplasmic translocation and inhibits their cytoplasmic replication by targeting them for ubiquitination-dependent destruction (99). Importantly, this would only target non-enveloped viral particles like noroviruses, rotaviruses, or human papilloma viruses, but not enveloped viruses, as these shed their opsonized envelopes on cytoplasmic entry. Recently, James and colleagues describe TRIM21 to recognize other isotypes as well, like IgM, and triggering a surge of proinflammatory cytokines, also in response to Ig-opsonized intracellular bacteria like *Salmonella* (101). It is unclear how the relative importance of this pathway in host-protect is, given that systemic elimination by other pathways (e.g., complement, FcγR, and myeloid system). The current evidence therefore suggest TRIM21 to be particularly important at locations where complement and the myeloid system are less prominently present, e.g., at mucosal surfaces of the gut. The relative importance of this system compared to the other receptor systems for viral elimination awaits further confirmation.

## Conclusion

Recent advances in knowledge of mucosal antibodies and novel receptor functions, provides a platform for work toward the induction of protective mucosal immune responses. Considerably further research into the precise mechanisms involved are required in order to understand how to elicit protective immune responses required for both systemic and mucosal protection, information that will also benefit passively administered immunoglobulin therapies.

## Conflict of Interest Statement

The authors declare that the research was conducted in the absence of any commercial or financial relationships that could be construed as a potential conflict of interest.
